# Mycotoxin detection in food and feed: bridging conventional analytical methods with emerging sensor-based technologies

**DOI:** 10.1007/s12550-026-00657-x

**Published:** 2026-06-15

**Authors:** Viola O. Okechukwu, Oluwasola A. Adelusi, Temitope R. Fagbohun, Okechukwu J. Okonkwo, Patrick B. Njobeh

**Affiliations:** 1 Department of Biotechnology and Food Technology, Doornfontein Campus, Faculty of Science, University of Johannesburg, P.O. Box 17011, Johannesburg, South Africa; 2 Department of Environmental, Water and Earth Sciences, Faculty of Science, Tshwane University of Technology, 175 Nelson Mandela Drive, Pretoria Central, 0001 South Africa

**Keywords:** Mycotoxins, Risk assessment, Emerging sensor technologies, Food safety, Nanomaterial-based sensors, Detection techniques

## Abstract

Mycotoxins, a harmful secondary metabolite released by filamentous fungi, are a major and frequent contaminant of agricultural products. They pose a major threat to food and feed security, public health, and global trade. Major mycotoxins of interest include aflatoxins, ochratoxin A, trichothecenes, fumonisins, zearalenone, patulin, deoxynivalenol, and citrinin, are associated with severe toxicological effects such as hepatocarcinogenicity, nephrotoxicity, immunosuppression, and endocrine disruption. A wide range of analytical methods has been developed for their detection, ranging from conventional chromatographic and spectroscopic techniques to biosensors Although conventional techniques offer high accuracy and sensitivity, their dependence on costly instrumentation, lengthy analysis procedures, and specialized laboratory infrastructure limits their suitability for routine or field-level monitoring. To address these challenges, recent research has increasingly focused on sensor-based technologies, electrochemical, optical, piezoelectric, electronic-nose, and solid-state gas sensors, which offer rapid, portable, and cost-effective alternatives. This review not only synthesizes these advances but also introduces a distinctive emphasis on volatile organic compounds (VOCs) as emerging, non-invasive biomarkers for early fungal contamination detection, a perspective rarely highlighted in existing literature. Together, these developments offer promising pathways toward robust, real-time surveillance tools that strengthen food and feed safety. This review also discussed their applications, limitations, and future perspectives are also discussed.

## Introduction

Mycotoxins, which are toxic metabolites produced by members of the fungal genera Aspergillus, Penicillium, and Fusarium, contaminate and adulterate agricultural products during cultivation (pre-harvest) or storage (post-harvest) (Gurikar et al. 2023). Pre-harvest contamination is induced by environmental stress factors such as high temperatures, drought, humidity, and insect infestation, which promote fungal colonization. For example, Fusarium species infest maize, wheat, and barley in temperate regions, producing fumonisins (FB_1_, FB_2_, FB_3_), deoxynivalenol (DON), and zearalenone (ZEN) (Munkvold 2016; Bryła et al. 2022). Post-harvest contamination results from delayed drying, poor aeration, and high humidity, favouring xerophilic fungi such as Aspergillus and Penicillium, which are responsible for aflatoxin(AFs) and ochratoxin production (Gurikar et al. 2023). In addition, contaminated feed facilitates the carryover of mycotoxins into animal products; for instance, AFB_1_ is biotransformed into AFM_1_ in milk, presenting an additional risk to consumers.

The global prevalence of mycotoxins in food and feed poses a significant risk to food safety, public health, and international trade. Cereal-based products such as maize, wheat, barley, and rice are frequently affected, with aflatoxins dominating in tropical and subtropical regions and DON and ZEN widespread in temperate climates (Gurikar et al. 2023). Ochratoxin A (OTA), on the other hand, contaminates a variety of products including coffee, dried fruits, grapes, wine, and cereals (Thompson and Darwish 2019; González-Curbelo and Kabak 2023). These contaminants often co-occur in a single matrix, complicating analytical detection and increasing the likelihood of synergistic toxicity (Thompson and Darwish, 2019). Climate change further intensifies these risks by influencing fungal distribution and seasonal growth patterns, while inadequate hygiene and weak infrastructure lead to cross-contamination during harvesting, processing, and storage, especially in developing regions (Dövényi-Nagy et al. 2020; Mahunu et al. 2024). Reports indicate that 60–80% of global agricultural products contain at least one regulated mycotoxin (Gurikar et al. 2023).

Mycotoxins exert a wide range of toxic effects, including hepatotoxic, nephrotoxic, immunosuppressive, and endocrine-disrupting impacts, depending on the type of toxin and level of exposure (Gurikar et al. 2023). These toxic effects emphasize the urgent need for effective monitoring and stringent regulatory measures to reduce food safety and public health risks. While the challenges and impacts of mycotoxin contamination are well documented, an equally critical issue is the need for advanced detection technologies capable of rapid, sensitive, and field-deployable monitoring. Conventional analytical methods such as chromatographic, spectroscopic, and immunochemical, remain the reference standards but are often time-consuming, expensive, and heavily dependent on laboratory infrastructure (Okechukwu et al. 2024a, c). These limitations highlight the growing need for sensor-based technologies that offer real-time, portable, and cost-effective detection suitable for diverse agricultural environments.

This review provides a comprehensive synthesis that bridges conventional analytical techniques with nanomaterial-based sensor technologies for mycotoxin detection in food and feed. Unlike existing reviews that discuss these approaches separately, this work combines chromatographic, spectroscopic, and immunochemical methods with nanomaterial‒enhanced electrochemical, optical, and piezoelectric sensors. A distinctive feature of this review is the inclusion of volatile organic compounds (VOCs) as indirect biomarkers of fungal contamination, offering a novel, non-invasive route for early detection using electronic-nose and solid-state gas sensors. By consolidating advances and critically evaluating detection performance, material innovations, sustainability, and applicability particularly in low-resource agricultural settings this study establishes a clear rationale for next-generation, sensor-driven mycotoxin monitoring. It thereby advances both conceptual and applied insights into nanotechnology-based and environmentally sustainable strategies for global food safety.

### Mycotoxin toxicological effects and relevance of regulatory standards

Mycotoxins exhibit a range of toxicological effects in humans and animals, influenced by their chemical structure, exposure level, duration, and host susceptibility (Chilaka et al. 2022; Misihairabgwi et al. 2019). Among these, AFs, especially AFB_1_, are highly potent hepatotoxins and carcinogens that form DNA adducts, leading to mutations and hepatocellular carcinomas. They are classified as Group 1 human carcinogens by the International Agency for Research on Cancer (IARC) (Okechukwu et al. 2024a, b). OTA, another prevalent mycotoxin, exerts nephrotoxic, teratogenic, and immunosuppressive effects, and has been associated with Balkan endemic nephropathy (Więckowska et al. 2024). FBs, especially FB_1_, disrupt sphingolipid metabolism, causing neural tube defects in humans and leukoencephalomalacia in horses. Other significant mycotoxins include deoxynivalenol, which inhibits protein synthesis and induces gastrointestinal disturbances and growth impairment, and ZEN, which exhibits estrogenic effects and leads to reproductive disorders in farm animals (Hussein and Brasel 2001).

To safeguard public health, regulatory authorities such as the European Food Safety Authority (EFSA), the Codex Alimentarius Commission, and the U.S. Food and Drug Administration (FDA) have established maximum permissible levels for key mycotoxins, generally ranging from sub-ppb to low µg/kg levels, depending on the toxin and food matrix (López-García 2022; Wu 2004). These stringent limits highlight the toxicological significance of mycotoxins and establish essential benchmarks for analytical and sensor-based detection technologies, which must achieve extremely low detection limits and high selectivity to ensure compliance with food safety standards. The increasing challenge of the co-occurrence of multiple mycotoxins, often exhibiting additive or synergistic toxicities, further highlights the need for advanced, sensitive, and rapid detection systems to enable early identification and effective risk mitigation (Karsauliya et al. 2022; Awuchi et al. 2021).

### Conventional methods for detecting mycotoxins and their limitations

Given the frequent occurrence of mycotoxins in food and feed and their toxic effects on human and animal health, accurate and timely detection of these contaminants is essential to ensure food safety and meet regulatory standards. Unfortunately, mycotoxins are typically present in agricultural products at trace levels, often in the µg/kg or nanogram range, making their detection challenging (Agriopoulou et al. 2020). This has led to the development of a range of analytical methods to meet the growing demand for a reliable, rapid, and cost-effective mycotoxin analysis in food safety and quality control. Currently, chromatographic techniques are the most widely utilized methods for mycotoxin analysis in agricultural products (Singh and Mehta 2020; Okechukwu et al. 2024a). Thin layer chromatography (TLC), the earliest chromatographic technique, is commonly used in many laboratories as a quick visual tool for mycotoxins. However, advances in mycotoxin analysis emphasize the need for reliable, rapid, user-friendly, and less expensive methods that are capable of simultaneously detecting and quantifying multiple mycotoxins with high sensitivity and selectivity in a single analytical procedure (Okechukwu et al. 2024a).

To address these analytical demands, several chromatographic techniques have been developed, including high-performance liquid chromatography (HPLC) coupled with detectors such as fluorescence (FLD), ultraviolet (UV), diode array (DAD), and mass spectrometry (MS). In addition, ultra-high-performance liquid chromatography (UHPLC) utilizing columns with particle sizes of less than 2 μm provides improved resolution and faster analysis (Agriopoulou et al. 2020). Gas chromatography (GC), combined with detectors such as flame ionization (FID), electron capture (ECD), or mass spectrometry (MS), has also been used for the quantification and detection of mycotoxins. However, a derivatization step is often necessary since many mycotoxins exhibit high polarity and low volatility, which limits the routine use of GC in mycotoxin analysis. The integration of liquid chromatography with mass spectrometry (LC-MS) has significantly improved the accuracy and sensitivity of mycotoxin analysis.

HPLC and HPLC-FLD are widely used for food analysis, while TLC and other low-resolution methods are becoming less common due to their lower analytical performance (Okechukwu et al. 2024a). Chromatographic methods, such as LC and GC are highly accurate and sensitive and can detect multiple analytes. However, they require complex and time-consuming preparation steps, sophisticated instrumentation, and specialized technical expertise (Okechukwu et al. 2024a; Dai et al. 2025; Man et al. 2017). Their reliance on expensive reagents, labour-intensive procedures, and laboratory operation limits their practicality for on-site testing of mycotoxin, especially at low concentrations (Man et al. 2017; Nolan et al. 2019). Additionally, the need for solvents and chemicals raises concerns about environmental impact and user safety. Despite these limitations, chromatographic methods remain essential for regulatory compliance and reference testing.

Furthermore, immunochemical methods, including lateral flow immunoassay (LFIA), enzyme-linked immunosorbent assay (ELISA), radioimmunoassay (RIA) and immunoaffinity chromatography (IAC) are widely used for detecting mycotoxins such as AFs, DON, OTA, ZEN, and FBs in food and feed (Deng et al. 2025). These methods utilize antigen-antibody binding reactions for sensitive and selective quantification. ELISA is commonly used for routine screening of contaminated samples due to its relative ease of use, cost-effectiveness, and capacity for high-throughput testing. LFIAs are portable and suitable for on-site use (Okechukwu et al. 2024a; Singh and Mehta 2020), offering qualitative or semi-quantitative results within minutes. IAC provides efficient cleanup and enrichment before chromatographic confirmation, while RIA, although highly sensitive for trace mycotoxin levels, is now less commonly used due to safety regulatory limitations (Agriopoulou et al. 2020). However, immunoassays have limitations such as cross-reactivity, limited stability of antibodies, and matrix interference, which can compromise accuracy (Dey et al. 2023; Cavalera 2021). While ELISA and LFIA are ideal for rapid screening of mycotoxins, IAC offers selective sample cleanup for chromatographic analysis.

Spectroscopic techniques such as UV–Vis, fluorescence, FTIR, NIR, and Raman spectroscopy are widely used for the qualitative and quantitative detection of mycotoxins in food and feed, based on their characteristic light absorption, emission, or vibrational properties (Deng et al. 2025). These methods provide rapid, non-destructive, and sensitive analysis, enabling the identification of molecular fingerprints associated with specific mycotoxins, including AFs, OTA, and ZEN (Deng et al. 2025). Their performance is influenced by factors such as instrument calibration, wavelength precision, sample matrix composition, solvent effects, and mycotoxin photostability, all of which influence analytical accuracy and reproducibility (Dey et al. 2023). Key parameters that influence chromatographic, spectroscopic, and immunochemical methods of mycotoxin detection are summarized in Table 1. Additionally, nanomaterial-based sensor technologies have emerged as promising, sensitive, portable, and real-time detection methods that address the limitations of conventional mycotoxin assays and meet the growing need for efficient on-site monitoring to ensure food safety (Lin et al. 2024; Nath 2024).

**Table 1:** Factors affecting conventional methods for mycotoxin detection

Technique category	Key performance factors
Spectroscopic techniques (Near-IR, Fluorescence)	• Instrument maintenance and alignment
	• Operator skill for wavelength calibration, sample preparation, and data interpretation
	• Optical pathlength and cuvette quality
	• Humidity, dust, ambient light interference, pH, and buffer composition
	• Mycotoxin photostability and chemical structure
Chromatographic methods (LC-MS, HPLC, GC-MS, TLC)	• Ratio and polarity affecting elution and separation quality
	• Mycotoxin stability
	• Detector type and sensitivity
	• Column type, temperature, and stationary phase
	• Operator skill, instrument maintenance, and laboratory conditions
	• Extraction solvents and cleanup methods determining
Immunochemical methods (RIA, LFIA, ELISA. IAC)	• Antibody type and source
	• Assay conditions and reaction environment (pH, ionic strength, temperature, incubation time, blocking and washing efficiency)
	• Antibody affinity (binding strength)
	• Enzyme–substrate system (in ELISA)
	• Antibody stability and storage conditions

### Biosensor for mycotoxin detection

**Fig. 1:**
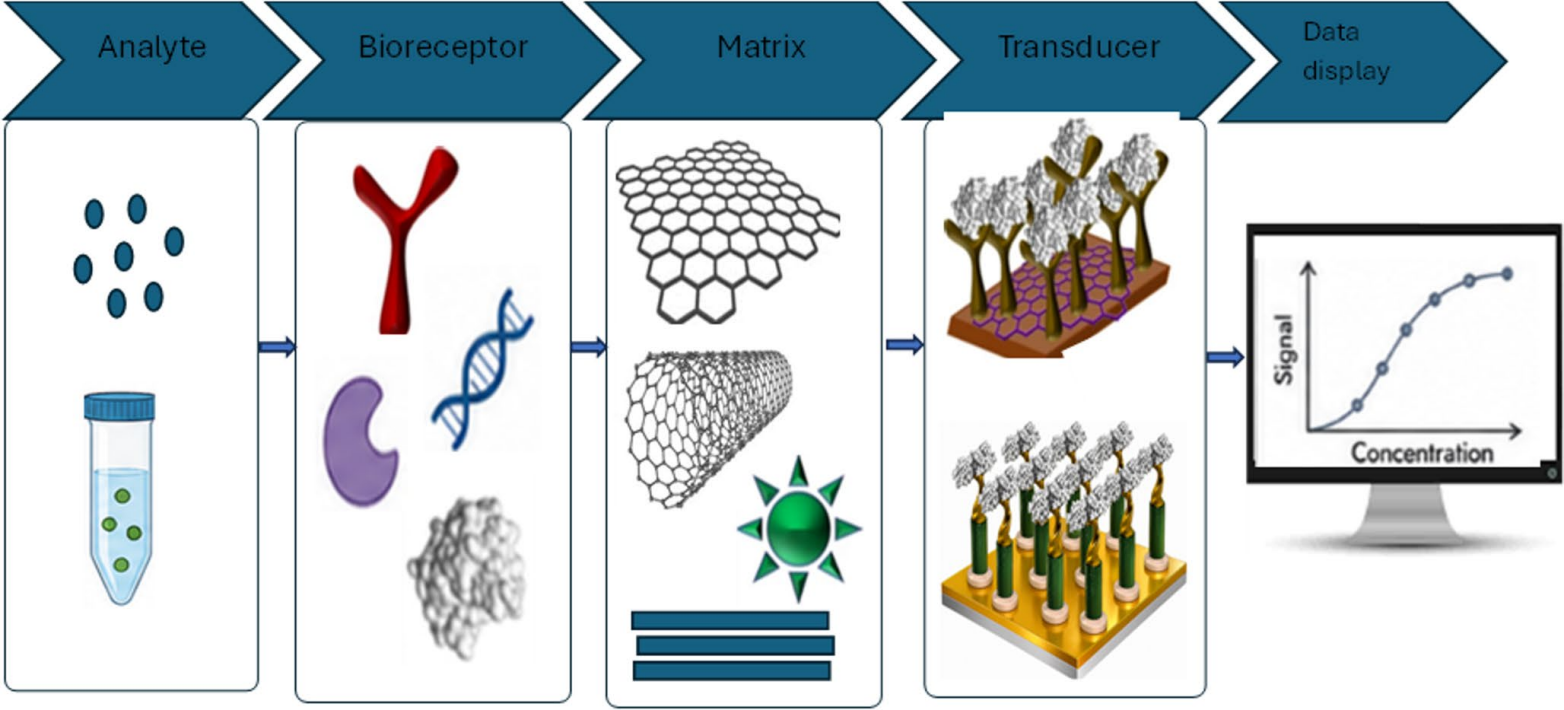
Structural representation of the key components in a biosensor

Biosensors are promising analytical tools for monitoring mycotoxins in food and feed matrices, combining high specificity and sensitivity with real-time, rapid screening capabilities (Szelenberger et al. 2024; Li et al. 2021b). These devices integrate a biological recognition element with a physicochemical transducer to form a unified system that translates specific molecular interactions into measurable signals (Fig. 1). The detection process begins with the target analyte, as mycotoxins are present in complex matrices such as cereals, nuts, and processed foods, thereby ensuring food safety and regulatory compliance. Biological recognition elements such as enzymes, antibodies, nucleic acid aptamers, whole cells, or molecularly imprinted polymers (MIPs) are selected based on their strong affinity and selectivity for specific mycotoxins (Kumar et al. 2022; Inshyna et al. 2020). The choice of the appropriate bioreceptor is crucial and depends on the complexity of the sample matrix, required sensitivity, and the physicochemical properties of the target analyte (Wang et al. 2022; Shrivastava and Sharma 2022). Each bioreceptor is designed to selectively identify and bind to its target analyte, thereby initiating a signal-generation process (Wang et al. 2022).

For instance, enzymes can catalyze reactions that produce electroactive or optically active products, while antibodies and aptamers rely on highly specific molecular recognition. MIPs, in contrast, offer synthetic binding sites with high stability and reusability (Saylan et al. 2024; Ali and Omer 2022). Following analyte recognition, a physicochemical change is induced, which is captured by the transducer and converted into a measurable signal (Saylan et al. 2024; Ali and Omer 2022). The transducer plays a central role in signal conversion, transforming the biorecognition event into detectable outputs that may be optical, electrical, mechanical, or thermal depending on the sensor design. The integration of a selective bioreceptor with a sensitive transducer ultimately determines the analytical performance of the biosensors (Saylan et al. 2024; Ali and Omer 2022). This coordinated interaction enables the detection of trace levels of mycotoxins, even in complex sample matrices, thereby enhancing sensitivity, selectivity, and reliability in mycotoxin analysis (Rathore et al. 2025).

Despite their advantages, biosensors face several limitations that hinder widespread application. These include limited stability and short shelf life of biological recognition elements, which are sensitive to environmental factors such as temperature, humidity, and pH (Kłos-Witkowska and Martsenyuk 2019; Demir et al. 2024). Matrix interference, non-specific adsorption, and signal drift can further reduce analytical accuracy. In addition, many biosensors exhibit limited reusability due to degradation of the bioreceptor layer during repeated use (Jia et al. 2024). The fabrication and integration process is complex, requiring careful immobilization of biological components while maintaining functionality and signal stability (Naresh and Lee 2021; Wu et al. 2023). Furthermore, the narrow optimal operating conditions and biofouling due to non-specific adsorption in complex samples can negatively affect performance and accuracy (Jiang et al. 2020; Mauriz 2020). However, biosensors offer significant advantages in terms of miniaturization, portability, and automation, especially when integrated with microfluidics and nanotechnology. These features make them indispensable for on-site, and real-time mycotoxin detection (Szelenberger et al. 2024). Further advancements, particularly in nanomaterial integration and sensor design, are expected to improve their stability, sensitivity and practical applicability in ensuring global food safety.

### Advances in the biosensor

Recent developments in biosensor technology have focused on enhancing performance through the integration of nanomaterials, miniaturization, and signal amplification techniques. Nanotechnology has significantly improved detection sensitivity and electron transfer while increasing the surface area available for bioreceptor immobilization. This enhancement is achieved by incorporating nanomaterials such as gold nanoparticles, carbon nanotubes, and quantum dots into sensor platforms (Kour et al. 2020; Kucherenko et al. 2019). These materials improve electron transfer rates, increase the surface area for bioreceptor immobilization, and amplify detection signals, thereby improving the sensitivity and lowering detection limits (Naresh and Lee 2021). Furthermore, the development of smartphone-integrated platforms and microfluidic systems has enabled fast, portable, and automated detection suitable for on-site applications (Nath 2024; Bueno et al. 2021). These advancements aim to address the limitations discussed in Sect. 1.4 and improve the reliability and practicality of biosensors in food safety monitoring.

## Sensor-based nanotechnology for mycotoxin detection

Nanotechnology, which involves the manipulation of materials at the nanoscale, has enabled the development of nanomaterials with unique physicochemical properties, including enhanced reactivity, high surface-to-volume ratios, and tunable optical and electronic characteristics (Khan et al. 2022; Rafeeq et al. 2025). These intrinsic properties have facilitated applications across diverse fields such as agriculture, food security, and environmental monitoring. The persistent issue of mycotoxin adulteration in food and feed has necessitated the need for advanced, sensitive, and rapid detection systems. In this context, nanotechnology-based methods have emerged as powerful tools for the detection of mycotoxins (Bueno et al. 2021). They enable the development of highly sensitive, portable, and low-cost sensors capable of identifying trace concentrations of mycotoxins in complex samples. Early and accurate detection is crucial to prevent the distribution of contaminated products, thereby reducing risks to public health and economic losses (Okechukwu et al. 2024a, b).

Recently, sensor techniques based on nanomaterials have attracted great interest as reliable alternatives to conventional analytical methods due to their exceptional sensitivity (Bueno et al. 2021). They are promising tools for improving mycotoxin detection in food and feed samples due to their excellent sensitivity and selectivity (Gao et al. 2021; Das and Mishra, 2022). These performance advantages arise directly from the physicochemical properties of the materials. For instance, an enhanced surface area improves bioreceptor immobilization density, leading to high signal output, while the catalytic and electronic attributes of metallic nanomaterials amplify electrochemical signals, thereby lowering detection limits. Nanomaterials are defined as substances with at least one dimension in the range of 1–100 nm or composed of nanoscale units arranged in a three-dimensional structure (Khan et al. 2022; Rafeeq et al. 2025). Due to their reduced size, these materials possess exceptionally high surface area-to-volume ratios, which impart unique physicochemical attributes compared to their bulk counterparts. These properties include improved adsorption capacity, enhanced catalytic activity and good electrical conductivity, all of which directly contribute to overall sensor performance, such as sensitivity and faster responses.

Various synthesis methods can be used to produce nanomaterials with controlled size, shape, and composition. These methods include top-down physical processes such as laser ablation, ball milling, sputtering, electron beam evaporation, and electrospraying. In contrast chemical bottom-up strategies involve sol-gel methods, hydrothermal synthesis, co-precipitation, and chemical vapor deposition, among others (Onyinye Okechukwu et al. 2024). These procedures yield a variety of nanostructures, including nanoparticles (NPs), metallic nanoparticles (MNPs), nanotubes (NTs), nanowires (NWs), nanorods (NRs), carbon allotropes, quantum dots (QDs), and composite nanostructures. The composition can encompass metals, metal oxides, dendrimers, mesoporous silica, micelles, liposomes, magnetic materials, polymers, metal-oxide frameworks (MOFs), and carbon-based materials, each offering specific advantages for targeted applications (Baccaro et al., Onyinye Okechukwu et al. 2024).

These sensors function through specific molecular interactions between nanostructured materials and mycotoxins, generating quantifiable signals that correlate with the concentration of the toxin (Lin et al. 2024; Sohrabi et al. 2022). The efficiency of this signal generation strongly depends strongly on the properties of the nanomaterial; for example, enhanced adsorption and binding affinity increase analyte capture efficiency, while improved conductivity and catalytic activity enhance signal transduction, resulting in lower limits of detection (LOD) and improved sensitivity. These characteristics make nanomaterials well-suited for extensive applications in agricultural and food processing environments (Okechukwu et al. 2024c). Compared to conventional chromatographic or immunoassay techniques, nanomaterial-based sensors offer faster response times and require minimal sample preparation (Chen and Inbaraj 2022). By utilizing the potential of sensor technologies, farmers, the food industry, and consumers can improve their ability to monitor mycotoxin contamination in food and intervene in time to prevent adverse health effects.

### Fundamentals of nanomaterials-based sensors

Advances in nanotechnology have significantly transformed sensors design, particularly in the highly sensitive detection of mycotoxins at low levels in complex food and feed matrices (Nihal et al. 2024). The incorporation of nanomaterials into sensors has revolutionized analytical detection systems by lowering detection limits, increasing sensitivity, and enabling real-time, on-site monitoring of trace contaminants such as mycotoxins. A typical sensor consists of three main components: a biological recognition element, a transducer, and a signal processing unit (Eddaif and Shaban 2023). Nanomaterials can be integrated into the transduction element or serve as an immobilization matrix for the biorecognition substance. Commonly used nanomaterials for detecting toxins and other analytes include metal oxide semiconductors (e.g., tin dioxide (SnO_2_), zinc oxide (ZnO), titanium dioxide (TiO_2_), nickel oxide (NiO), various carbon-based nanostructures such as carbon nanoparticles (CNPs), carbon nanotubes (CNTs), quantum dots, graphene oxide (GO), reduced GO), and conducting polymers along with their hybrid nanocomposites (Ayanda et al. 2024). These materials are chosen for their unique physicochemical properties including high conductivity, large surface-to-volume ratio, tunable surface chemical functionalities, and inherent chemical stability, all of which contribute to lower detection limits, faster response times, and improved sensor performance (Okechukwu et al., 2021, 2024b).

**Fig. 2:**
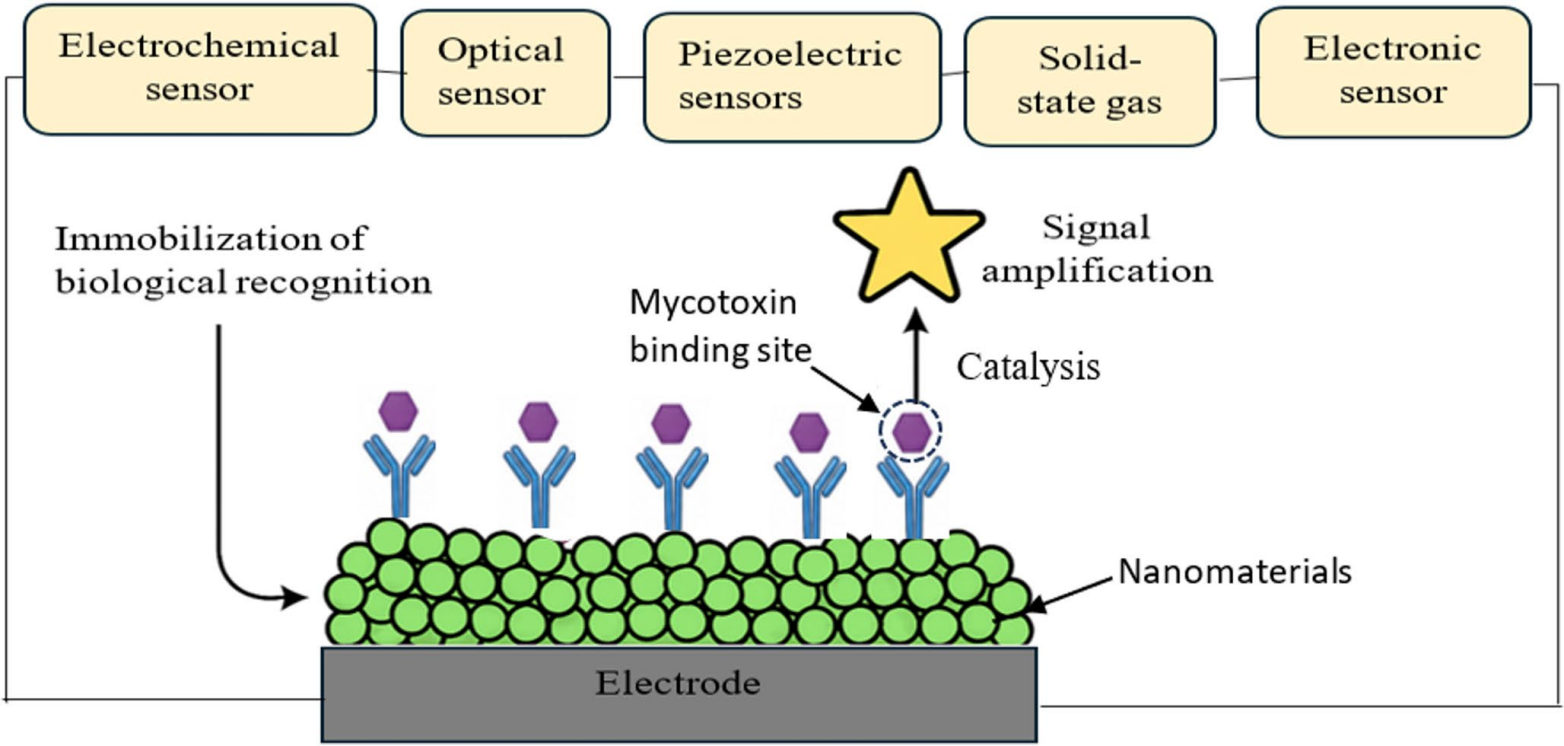
Typical diagram illustrating the deposition and integration of nanomaterials onto the electrode surface for selective mycotoxin detection

Signal transmission in nanomaterials-based sensors occurs through mechanisms such as electrochemical (e.g., current, potential, or impedance changes), optical (e.g., fluorescence, absorption, or plasmon resonance shifts), and piezoelectric (e.g., resonance frequency shifts due to mass loading) reactions (Yang et al. 2020; Selvolini and Marrazza 2023). In typical sensor fabrication (Fig. 2), nanomaterials are deposited onto the electrode surface, which serves as a signal conducting, and reactive interfaces. These surfaces provide sites for the immobilization of highly selective biorecognition elements, including antibodies, aptamers, or molecularly imprinted polymers (Kaur et al. 2023; Szelenberger et al. 2024).

Upon interacting with a specific mycotoxin, the recognition element undergoes a conformational or chemical change, resulting in a detectable physicochemical response. This interaction is converted into a quantifiable electrical or optical output signal, allowing for highly selective and sensitive detection (Frutiger et al. 2021; Wang et al. 2022). The principles behind various classes of nanomaterial-based sensors developed for mycotoxin detection are discussed in the following section. A comparative summary of these sensors’ applications and their analytical performance parameters in food and feed samples is presented in Table 2.

**Table 2:** Advances in nanomaterial-based sensor technologies for mycotoxin detection

Matrix	Method type	Analytical technique	Mycotoxin	Nanomaterial used	LOD	Linear range	Refences
Meat product	Electrochemical	DPV	FB1	AuNPs	0.08 ng/L	1 ng/L‒1 mg/L	(Zhao et al. 2024a)
Cereals	Electrochemical	DPV/EIS	AFB_1_ OTA	AuNPs/PEI-rGO/MOF	6.2 ng/L 3.7 ng/L	0.01‒1000 ng/mL	(XU et al. 2024c)
Rice flour	Electrochemical	DPV	AFB_1_	AUNPs	0.05	0.05–150 ng/mL	(Shi et al. 2020)
Rice, oats, wheat and barley	Optical	Fluorometry	OTA	Recombinant Fluonanobody Red QDs	5 pg/mL	5–5000 pg/mL	(Su et al.^2022^)
Cereals	Piezoelectric	QCM	AFB_1_	NiO/P5FIn	0.0015ng/ml	0.005‒50 ng/ml	(Lu et al. 2020)
Coffee samples	Electrochemical	EIS	OTA	Thin-film Au electrode	0.15 ng/mL	0.5 to 100 ng/mL	(de Oliveira et al. 2023)
Cereal products	Piezoelectric	QCM	ZEN	AuNPs decorated on the diazonium	0.182 ng/ml	0.1–250 ng/mL	(Gheni et al. 2025)
Grain samples (maize and oats)	Electrochemical	EIS	ZEN	AuNPs	1.1 ng/kg	10 ng/kg ‒ 10 mg/kg,	(Zhao et al. 2024b)
Peanut Maize	Optical	Colorimetry	AFB_1_	TiO_2_ NPs	1.4 µg/kg 0.3 µg/kg	1–5 µg/kg 1–20 µg/kg	(Khansili and Krishna 2022)
Roasted coffee & wine	Electrochemical	DPV/EIS	OTA	AuNPs	11 pg/mL	0.5‒70ng/mL	(Argoubi et al. 2024)
rice samples	Piezoelectric	QCM	ZEN	MoS_2_NPs-MWCNTs	0.30 ng/L	1.0–10.0 ng/L	(Çapar et al. 2023)
Red wine, Beer, Chinese liquor	Electrochemical	DPV	OTA	Au NPs/PIL-FMNS/CNT-MoS2/GCE	14 nM	0.5 − 15 µM	(Hu et al. 2022)
Apple juice	Optical	SPR	Patulin	PAT imprinted NPs	1.04 pg/mL	0.0001–10 ng/mL	(Çimen et al. 2022)
Milk	Electrochemical	DPV	AFB2	ZnO-NPs/CS/PPy	0.2 fg/mL	0.1‒1000 fg/mL	(El Hassani et al. 2020)
Husky rusky rice, peanut oil & wheat	Electrochemical	ASV	AFB_1_	Quantum dots	0.05 µg/kg	0.08–800 µg/kg.	(Xuan et al. 2020)
Herbs	Optical	SERS	AFB_1_	AuNPs	0.36 pg/mL	0.01‒100 ng/mL	(Song et al. 2022)
Herbs	Optical	SERS	OTA	AuNPs	0.034 pg/mL	0.001–10 ng/mL	(Song et al. 2022)
Food samples (corn, wheat flour, soy sauce, and milk)	Piezoelectric	QCM	ZEN	AuNPs	0.37 µg/L	nr	(Liu et al. 2021)
Breakfast cereal, rice flour, maize powder	Electrochemical	DPASV	ZEN	Cu-MOF/Fe3O4/GO	23.14ng/mL	159.2–2865.2 ng/mL	(Zeng et al. 2022)

*nr* not reported, *DPV *differential pulse voltammetry, *EIS* electrochemical impedance voltammetry

#### Electrochemical nanomaterial-based sensor

Electrochemical sensors have emerged as one of the most important platforms for mycotoxin detection due to their high sensitivity, specificity, portability, and affordability (Jiang et al. 2024). These devices convert biochemical interactions, typically between a biological recognition element (such as antibody, aptamer, enzyme, or MIP) and the target mycotoxin into electrical signals measured as current, potential, impedance, or conductivity (Wang et al. 2022; Nath 2024). Electrochemical sensors are classified into amperometric, potentiometric, conductometric, and impedimetric types depending on the nature of the electrical signal monitored (Nath 2024). Their simple electrode configuration, usually consisting of a working, reference, and counter electrodes, along with their potential for miniaturization, makes them particularly suitable for various applications (Onyinye Okechukwu et al. 2024).

Nanomaterials significantly enhance the analytical performance of these sensors by increasing the electrode surface area, accelerating electron transfer, and enabling higher immobilization efficiency of biorecognition molecules (Onyinye Okechukwu et al. 2024; Fritea et al. 2021). For example, gold nanoparticle/graphene-modified electrodes have been used for the ultrasensitive detection of aflatoxin B_1_ in the sub-nanogram range (Sultana 2019; Owino et al. 2008; Althagafi et al. 2019). Carbon-based nanomaterials facilitate rapid electron transfer and provide strong mechanical stability, while quantum dots offer electrochemiluminescent properties advantageous for hybrid electrochemical–optical sensing platforms. One of the major advantages of electrochemical sensors is their versatility across a wide range of food matrices. They have been successfully applied to detect mycotoxins in diverse commodities including cereals, nuts, dairy products, wine, and animal feed (Wang et al. 2022; Nath 2024). These sensors provide rapid response times, require minimal sample preparation, and can be fabricated as disposable strip-based devices or reusable electrodes (Dincer et al. 2019; Hassan et al. 2022).

Despite these advantages, several challenges still affect the performance of electrochemical sensors. Issues such as variability in electrode modification, limited long-term stability of immobilized biorecognition elements, and electrode fouling caused by complex food matrices can compromise analytical accuracy (Kamalasekaran and Sundramoorthy 2024). Additionally, interference from electroactive compounds present in samples must be minimized. Recent advances, including the use of aptamers, synthetic receptors such as MIPs, antifouling surface coatings, and improved electrode fabrication techniques, have been developed to address these limitations. Future developments in electrochemical mycotoxin sensing are expected to focus on integrating microfluidic systems for automated sample handling, embedding wireless communication modules for remote monitoring, and applying artificial intelligence for improved signal processing and pattern recognition (Jiang et al. 2024; Mutunga et al. 2024). These innovations, combined with the use of environmentally friendly materials and scalable manufacturing processes, promise to deliver robust, portable, and cost-effective sensing systems suitable for monitoring purpose (Pandhi et al. 2025).

#### Optical nanomaterial-based sensor

Optical sensors are sophisticated analytical instruments for mycotoxin detection that utilize the interactions between light and matter to achieve rapid, sensitive, and specific detection of target analytes (Szelenberger et al. 2024). When the target analyte binds to a recognition element, these sensors detect changes in optical characteristics such as absorption, fluorescence, refractive index, or Raman scattering (Hang et al. 2022; Madhu et al. 2023). Common types of optical sensors include surface-enhanced Raman scattering (SERS), surface plasmon resonance (SPR), and fluorescent-based sensors, each with different advantages and limitations. Fluorescence sensors can use either intrinsic fluorophores or fluorescent labels conjugated to biorecognition elements. When the mycotoxin binds to the recognition site, it alters the fluorescence intensity, lifetime, or emission wavelength, generating measurable signal (Wang et al. 2022). Quantum dots and upconversion nanoparticles have been used to improve fluorescence intensity and photostability, achieving extremely low detection limits (Wu et al. 2024; He and Yu 2024). For example, quantum dot-based sensors have successfully detected aflatoxin B_1_ in rice below the regulatory limits (Singh et al. 2022). Fluorescence resonance energy transfer (FRET) and “turn-on/turn-off” designs further increase sensitivity and enable multiplex detection.

SPR sensors provide label-free, real-time detection by measuring the changes in the refractive index at a metal surface (typically gold) when mycotoxins bind to immobilized receptors (Mahmoudpour et al. 2019; Wei et al. 2022). Light emitted from a source interacts with the sample or analyte, where it undergoes modulation through absorption, reflection, or scattering. The modified light is then detected by a photodetector, which converts these optical changes into an electrical signal proportional to the analyte concentration. SPR sensors are highly sensitive and capable of continuously monitoring molecular interactions, which is particularly useful for kinetic studies of the binding process (Wang et al. 2022). However, SPR instruments can be expensive and sensitive to environmental fluctuations, which limits their use in the field.

Recent advances aim to develop portable and cost-effective SPR sensors suitable for field deployment. SERS sensors amplify Raman scattering signals from molecules adsorbed on nanostructured metal surfaces, and generate characteristic spectral fingerprints of the target mycotoxins (Hang et al. 2022; Madhu et al. 2023). The high sensitivity of SERS enables the detection of single molecules in some cases, while its multiplexing capability allows for the simultaneous monitoring of multiple mycotoxins (Hassan et al. 2021). However, the reproducibility of SERS substrates and the complexity of spectral interpretation remain active research areas (Li et al. 2020). Recent progress in nanomaterials engineering has significantly improved the development of optical sensors, utilizing various classes of nanostructures such as metal nanoparticles, quantum dots, and plasmonic composites, tailored to improve selectivity, robustness, and sensitivity in mycotoxin detection.

Despite their excellent analytical performance, optical sensors face challenges related to cost, bulky instrumentation, and susceptibility to environmental influences (e.g., temperature and light intensity). To address these limitations, ongoing studies focus on miniaturization, designing robust portable devices, and incorporating of low-cost light sources and detectors (Hemida et al. 2023; Butt et al. 2024). Furthermore, coupling optical sensors with smartphones and microfluidic chips is emerging as a promising strategy to provide an accessible, user-friendly, and powerful platform for food safety monitoring.

#### Piezoelectric-based sensors

Piezoelectric sensors are mass-sensitive microelectrochemical devices that detect analyte–bioreceptor interactions by measuring the shifts in the resonance frequency of a quartz crystal (Benes et al. 1995; Kuchmenko and Lvova 2019). These sensors consist of a piezoelectric substrate coated with a selective recognition layer. When mycotoxins bind to this layer, the resulting mass change produces a proportional frequency shift, enabling real-time, label-free detection with high sensitivity (Wang et al. 2022; Szelenberger et al. 2024). Piezoelectric platforms are broadly classified into bulk acoustic wave (BAW) and surface acoustic wave (SAW) sensors. BAW devices, typically operating in thickness-shear or shear-horizontal modes, allow acoustic waves to propagate through the crystal, whereas SAW sensors detect Rayleigh waves traveling along the crystal surface.

The quartz crystal microbalance (QCM) is the most widely used piezoelectric sensor and can detect nanogram-level mass changes. QCM sensors functionalized with antibodies, aptamers, or MIPs have been applied to detect aflatoxins, OTA, and other mycotoxins, providing quantitative insights into binding interactions (Szelenberger et al. 2024; Xu et al. 2024b). These sensors are inherently simple, robust and relatively affordable compared to some optical methods. Platforms incorporating gold nanoparticles (AuNPs), magnetic nanocomposites (Au/Fe_3_O_4_), metal oxides such as NiO and ZnO NRs, CNTs, and MIPs exhibit enhanced sensitivity, broader detection ranges, and improved miniaturization potential. However, their performance is highly dependent on stable surface functionalization and environmental conditions, including humidity and temperature fluctuations, can introduce significant noise (Gulsaran et al. 2024). Non-specific binding and matrix interference also remain significant challenges (Chorsi et al. 2019).

Strategies such as antifouling coatings, optimized immobilization chemistry, and rigorous surface cleaning can improve selectivity and stability (Jiang et al. 2020; Banerjee et al. 2011). Advances in microfluidic integration and signal processing further expand their potential in food safety testing (Gao et al. 2020). Future development of piezoelectric sensors is likely to focus on multiplex detection, hybrid sensor formats, and wireless data transmission, making piezoelectric sensors well suited for real-time monitoring of contamination in storage and processing environments (Sobhan et al. 2025).

#### Performance evaluation parameters and critical comparative analysis of sensor performance with LC-MS

The performance of nanomaterial-based sensors for mycotoxin detection is typically reported using analytical metrics such as LOD, sensitivity, selectivity, and linear dynamic range. While these parameters describe analytical capability, they do not fully capture real-world performance, particularly when compared with established reference methods such as LC-MS/MS (Ciko et al. 2024). A more meaningful evaluation requires consideration of additional factors, including operational reliability, matrix tolerance, reproducibility, and validation status Table 3. Therefore, sensor performance should be assessed using broader dimensions such as robustness, accuracy, stability, and adaptability. The robustness of a sensor refers to its ability to maintain performance in the presence of matrix and environmental interferences, which are widely reported challenges in complex food systems (Nihal et al. 2024).

**Table 3:** Comparative evaluation of LC̶ MS and nanomaterial-based sensor technologies for mycotoxin detection

Parameters	LC – MS	Electrochemical sensor	Optical Sensors	Piezoelectrical sensors	Electronic nose	Solid state gas sensors
Reproducibility	Very high	Moderate	Moderate	Moderate	Moderate	Moderate
LOD	Very low	Very low (ng/L)	Very low (fg–pg/L)	Low to moderate	Moderate	Moderate
Cost per analysis	High	Low	Moderate-High	Moderate	Moderate	Moderate
Sensitivity	Very high	High	High	Low-moderate	Moderate	Moderate
Matrix tolerance	Low	High	Moderate –high	High	High	High
Selectivity	Very high	High	High	Moderate	Low ‒moderate	Low ‒moderate
Stability	High	Limited	Moderate	Moderate	Moderate	Moderate
Portability	Very low	High	Moderate	Moderate	High	High
Response time	Longer time	Very fast	Very fast – Moderate	Moderate	Rapid	Rapid
Validation status	Fully validated	Limited	Limited	Limited	Limited	Limited
Multiplex detection	Excellent	Limited – moderate	High	Limited	Limited	Limited
Field application	Poor	Excellent screening	Moderate	moderate	Non-invasive screening	Continuous monitoring

Sensor repeatability describes the accuracy and consistency of measurements under varying conditions, while stability refers to performance decline over time due to factors such as surface fouling, signal drift, or instability of the recognition element (Nihal et al. 2024). Within this framework, the frequently reported ultra-low LODs of nanomaterial-based sensors should be interpreted cautiously. For example, although electrochemical sensors have reported LODs as low as 0.08 ng/L for fumonisin B1, LC-MS/MS methods typically achieve comparable sensitivity with significantly higher robustness, reproducibility, and validated performance across complex matrices such as maize, feed, and dairy products. Moreover, LC-MS/MS provides reliable quantification with minimal matrix interference through standardized sample preparation and calibration protocols, whereas sensor-based systems are often more susceptible to matrix effects, including non-specific binding, signal drift, and interference from co-existing compounds (Hu et al. 2023; Jing et al. 2024).

A critical limitation of sensor technologies lies in their variability and lack of standardization. Sensor-to-sensor reproducibility, long-term stability, and batch consistency remain major challenges, particularly for devices incorporating biological recognition elements such as antibodies or enzymes. Environmental factors, including temperature, humidity, and sample heterogeneity, further affect sensor performance, limiting their reliability in field applications (Ariyaratne et al. 2023). Additionally, most sensor platforms lack comprehensive validation against internationally accepted regulatory standards, which restricts their adoption for routine compliance testing (Ariyaratne et al. 2023; Nihal et al. 2024).

Despite these limitations, nanomaterial-based sensors offer clear advantages, including rapid response, portability, cost-effectiveness, and suitability for on-site screening. As such, they are best positioned as complementary tools within a tiered detection framework, where rapid on-site screening is followed by confirmatory analysis using validated methods such as LC-MS/MS. This integrated approach balances speed and practicality with analytical reliability, supporting more efficient and accurate mycotoxin monitoring (Ciko et al. 2024).

## Emerging sensor techniques for the indirect detection of mycotoxins using volatile organic compounds (VOCs) from fungi

Recent advances in materials science, nanotechnology, and data-driven analytics have significantly accelerated the development of VOC-based sensing as an alternative to conventional mycotoxin detection approaches. VOCs are low-molecular-weight metabolites that can readily detected in the headspace of contaminated products, enabling non-invasive and real-time monitoring of fungal contamination (Khan et al. 2024). Fungal VOCs comprise a diverse group of compounds, including alcohols, ketones, aldehydes, hydrocarbons, phenols, heterocyclic compounds, sulphur-containing derivatives, and terpenes, produced during fungal metabolism. Their composition varies with fungal species, substrate type, growth stage, and environmental conditions, resulting in highly dynamic and matrix-dependent emission profiles (Cosetta et al. 2020; Kowalska et al. 2022). Several studies have demonstrated associations between specific VOCs and mycotoxin production.

For instance, Ji et al. (2022) reported that Fusarium graminearum emits specific VOC profiles that vary by growth stage and substrate, correlating with zearalenone production. Similarly, Li et al. (2021a) identified compounds such as oct-1-en-3-ol, 2-butanone, benzaldehyde, and 2-pentyl furan, as dominant during the later stages of fungal growth. These findings suggest that VOC emissions evolve over time and may indicate different phases of fungal metabolism, including the transition from primary growth to secondary metabolite (mycotoxin) biosynthesis. Also, YIN et al. (2026) reported that Penicillium expansum and Penicillium polonicum strains produce diverse and distinct VOC associated with differences in toxicity. Their finding indicated the functional relevance of VOC composition in fungal behaviour.

At the biochemical level, VOC production is closely linked to fungal metabolic pathways that are also involved in mycotoxin biosynthesis. Recent studies show that both VOCs and mycotoxins originate from shared intermediates, such as acetyl-CoA and polyketide precursors, linking primary metabolism with secondary metabolite production (Zhu et al. 2026; Amuzu et al. 2025; Huffman et al. 2010). VOCs, such as alcohols, ketones, and terpenes, are produced via fatty acid oxidation and terpenoid pathways, which are metabolically connected to toxin biosynthetic routes. These pathways are further regulated by global transcriptional regulators (e.g., LaeA, VeA), which control the activation of secondary metabolism and contribute to the observed decoupling between VOC production and mycotoxin biosynthesis (Amuzu et al. 2025).

However, VOC emissions are not quantitatively correlated with mycotoxin levels. VOCs are typically produced during early growth phases and signify general metabolic activity, whereas mycotoxins are synthesized later under specific stress conditions, such as changes in water activity, temperature, or nutrient limitation. As a result, VOC signals often precede detectable toxin accumulation, making them effective early-warning indicators but unsuitable as reliable quantitative proxies for mycotoxin concentration (Zhu et al. 2026). Recent experimental studies further confirm that VOC patterns can indicate fungal presence even when mycotoxin concentrations remain below regulatory thresholds (Zhu et al. 2026; Moore et al. 2021). Another critical limitation highlighted in recent research is the lack of specificity in VOC profiles. Multiple fungal species, including toxigenic and non-toxigenic strains, can produce overlapping VOCs such as 1-octen-3-ol, 3-octanone, and 2-pentylfuran, particularly during early growth stages (Li et al. 2021a; Zhu et al. 2026). Furthermore, according to Gunda et al. (Gunda et al. 2026), VOC emissions are strongly influenced by environmental conditions and substrate composition, leading to high variability across different food and feed matrices. In real-world storage environments, such as grain silos, VOC mixtures originate from fungi, plant materials, microbial communities, and environmental contaminants, complicating signal interpretation.

Electronic noses and MOS sensors lack molecular specificity and produce composite signals, necessitating chemometric analysis (Berna 2010). However, their performance is affected by humidity, temperature fluctuations, and sensor drift, reducing reliability in complex environments (Okechukwu et al. 2024a). These interferences can obscure subtle VOC changes associated with fungal contamination, further limiting the reliability of quantitative analysis. An integrated approach combines rapid VOC-based sensor for high-throughput screening of agricultural products with confirmatory analysis using conventional methods such as LC-MS or HPLC-MS for accurate mycotoxin quantification. This tiered system improves efficiency and reduces costs, as sensor- based devices act as early warning tools for potential contamination, while chromatographic or selective biosensor techniques offer accurate toxin measurement.

### Electronic-nose sensor (E-nose sensor)

E-nose sensors are emerging analytical devices that combine cross-reactive chemical sensor arrays with advanced pattern-recognition algorithms to detect and classify complex gas mixtures. They hold significant potential for food safety evaluation and environmental monitoring, particularly for rapid and non-destructive detection of mycotoxin contamination (Okechukwu et al. 2024a; Leggieri et al. 2021). Modelled after the human olfactory system, e-nose devices use broadly responsive sensors coupled with computational tools to distinguish VOC profiles associated with toxigenic fungi (Sanislav et al. 2025; Singh et al. 2025; Bonah et al. 2020; Loulier et al. 2020). Unlike conventional analytical methods, which are laborious and laboratory-dependent, e-nose systems enable real-time, on-site analysis of VOCs that indicate fungal growth, spoilage, or contamination (Bonah et al. 2020; Loulier et al. 2020). Since their introduction in 1982, continuous improvements in sensor materials, signal processing, and machine-learning algorithms have enhanced their performance (Alfieri et al. 2024; Meher and Zarouri 2025).

E-nose technologies are particularly effective for the early detection of mycotoxin-producing fungi because they recognize characteristic VOCs produced during fungal metabolism, often before significant toxin accumulation occurs (Suresh et al. 2025; Hamad et al. 2023; Neme and Mohammed 2017). Their usefulness in bulk commodities, such as grains and nuts, is noteworthy, as traditional sampling methods may overlook localized contamination. When exposed to VOCs, sensor arrays generate response patterns that are processed using multivariate statistics or machine-learning models to classify samples and, in some cases, estimate contamination levels (Zhai et al. 2024). Successful applications include detecting A. flavus in maize and peanuts and Penicillium species in coffee and grapes.

Despite their promise, e-nose systems still face challenges. Sensor drift can affects long-term stability, while humidity, temperature, and inconsistent headspace conditions may reduce reproducibility across different commodities (Wysocka and Dębowski 2025) Sensitivity and specificity can also vary, particularly when distinguishing closely related odour profiles. Current research focuses on enhancing sensor selectivity, stability, and portability, as well as incorporating artificial intelligence (AI) and machine learning to improve pattern recognition accuracy (Wysocka and Dębowski 2025; Singh et al. 2025). Hybrid systems that integrate e-nose technology with techniques such as GC-MS, hyperspectral imaging, or FTIR spectroscopy offer improved detection reliability through multimodal data fusion (Visconti et al. 2023). Ensuring long-term performance requires robust calibration using reference methods like LC-MS, drift-correction algorithms, and standardized sampling protocols (Vanaraj et al. 2025). With continued advancements, e-nose systems are expected to become integral tools for real-time mycotoxin monitoring, contributing to improved food safety and public health protection (Singh et al. 2025; Vanaraj et al. 2025).

### Solid-state gas sensor

Solid-state gas sensors represent a promising and rapidly advancing technology for the indirect detection of mycotoxins through the monitoring of VOCs emitted by toxigenic fungi (Garbacz et al. 2020; Poeta et al. 2023). Mycotoxigenic species such as A. flavus, F. verticillioides, and P. verrucosum release specific VOCs during the colonization of crops and food matrices, which can serve as reliable biomarkers for fungal growth and potential toxin presence. These sensors, typically based on MOS materials, detect fungal VOCs by measuring changes in electrical resistance or conductivity (Tomić et al. 2021). This method enables early, non-destructive monitoring of contamination in storage facilities, silos, and processing environments, providing a valuable alternative to conventional analytical methods (Okechukwu et al. 2024c; Xu et al. 2024a).

**Fig. 3:**
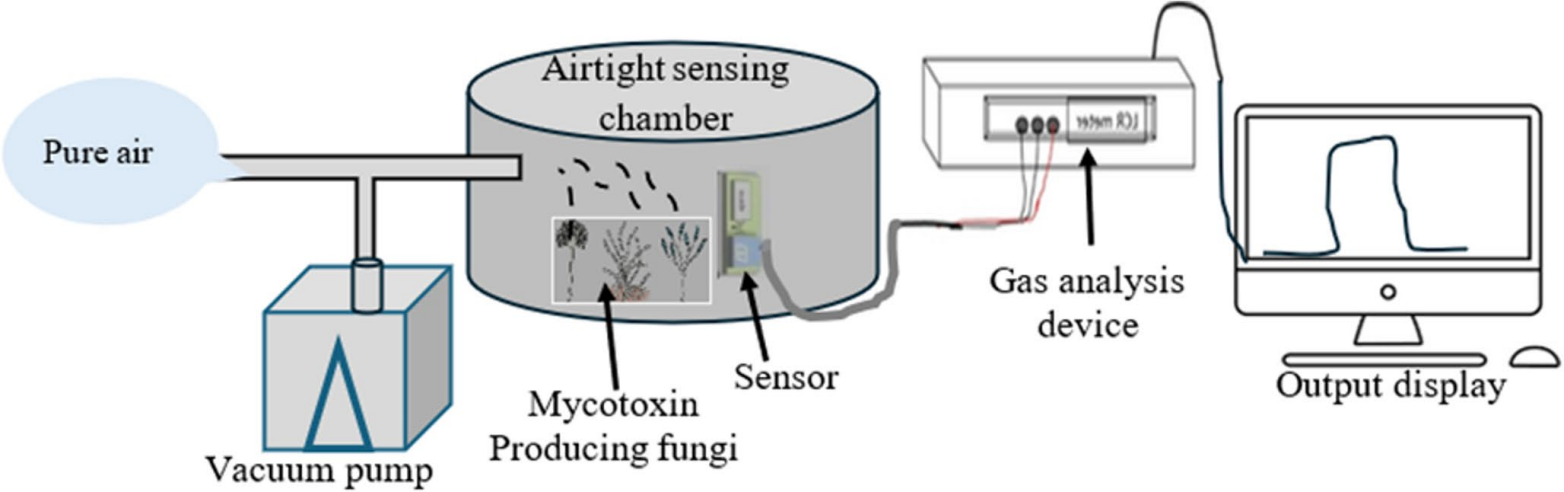
Typical illustration of a VOC-based solid-state gas sensing system for indirect detection of mycotoxin

The sensing principle relies on the interaction between VOC molecules and the surface of the active sensing layer (Okechukwu et al. 2019, 2021). Oxygen species adsorb onto the sensor surface and capture electrons from the conduction band at room or elevated temperatures, forming a surface depletion layer that increases electrical resistivity (Okechukwu et al. 2021). Figure 3 presents the sealed sensing chamber equipped with a gas sensor connected to a gas analysis device and a mobile computer for signal acquisition and output display. When VOCs emitted by toxigenic fungi interact with adsorbed oxygen species on the surface of the MOS, electrons are released into the conduction band, resulting in a measurable change in electrical resistance proportional to the VOC concentration (Onyinye Okechukwu et al. 2024; Malepe et al. 2022). Different VOCs produce distinct reaction patterns depending on their reactivity and adsorption kinetics, allowing the differentiation between fungal species and contamination levels (Meena et al. 2021).

Nanostructuring metal oxides into the form of rods, nanowires, or nanoparticles significantly enhances sensor performance by increasing surface area and the number of active adsorption sites (Okechukwu 2018; Kaur et al. 2022). Functionalization with other nanomaterials, such as GO, noble metal nanoparticles (e.g., Au, Pt), or polymers, further improves sensitivity, selectivity, and stability. For example, SnO_2_–GO hybrid sensors have shown improved responses to VOCs emitted by A. flavus, enabling room-temperature detection of fungal contamination (Okechukwu et al. 2024c). Similarly, selective coatings or catalytic metal doping enhance target specificity for VOCs and minimize interference from ambient gases, such as water vapour or CO_2_ (Feng et al. 2023; Chu et al. 2022).

A main advantage of solid-state gas sensors is their ability to detect contamination at an early stage, often before visible mould growth or significant mycotoxin accumulation occurs (Garbacz et al. 2020). This early-warning capability is critical for proactive management of stored agricultural products, allowing timely interventions to prevent spoilage and economic losses [168, 169]. These sensors are compact, cost-effective, and scalable, making them suitable for continuous real-time monitoring under field conditions (Okechukwu et al. 2024c). When used in arrays, they offer enhanced selectivity and can be integrated with pattern-recognition algorithms to distinguish VOC profiles from different fungal species or strains (Arora et al. 2022).

Despite these strengths, several challenges still limit the practical application of these sensors for routine mycotoxin monitoring. Environmental factors such as humidity, temperature, and the presence of non-specific VOCs can affect sensitivity and accuracy, while long-term stability and reproducibility remain major concerns (Wei et al. 2023; Kannan et al. 2024). Addressing these limitations requires advanced material engineering, robust calibration against reference methods, and the incorporation of intelligent data-processing algorithms. Coupling gas sensors with wireless communication and cloud-based data analytics could further enhance their role in smart food safety systems (Wei et al. 2023; Kannan et al. 2024). Continued research into the fungal VOC profiles, improved nanomaterial design, and hybrid sensing approaches is expected to yield reliable, field-ready gas sensors for effective mycotoxin risk assessment (Kannan et al. 2024). Various applications of electronic-nose and solid-state gas sensors for mycotoxin detection using fungal VOCs are summarized in Table 4.

**Table 4:** Few studies on electronic-nose and solid-state gas sensors for indirect detection of mycotoxins through volatile organic compounds (VOCs) emitted by toxigenic fungi

Matrix	Mycotoxin	Method type	Nanomaterial used	LOD	Accuracy (%)	References
Maize	AFs	E-nose	Fox 3000	10 µg/kg	76–79%	(Machungo et al. 2022)
Wheat	AFs	E-nose	DiagNose	‒	81–94%	(Machungo et al. 2022)
Cereal	AFs	E-nose	Cyranose 320	‒	68–75%	(Machungo et al. 2022)
Wheat flour	DON	Solid state	Fe_3_O_4_, NH_2_-MWCNTs	0.24 ng/mL	nr	(WEI et al., 2023)
Water	AFB_1_	Solid state	pNAGA	10 µg/kg	nr	(SZLAG et al. 2019)
Not specified	AFB_1_	Solid state	ZrO_2_@RGO	2.54 ng/mL	nr	(Chauhan et al. 2022)
Red meat degradation	DON	Solid state	ZnO\WSe_2_	‒	nr	(SIRAJ et al. 2025)
Milk	AFM_1_	Solid state	MIPs (AuNP based)	0.4 pg/mL	nr	(Akgönüllü et al. 2021)
Dry-cured meat	OTA	E-nose	MOS	‒	98–88%	(Lippolis et al. 2016)
Wheat	AFB_1_	E-nose	MIP@CdTe QDs	4 ng/g,	99–102	(Guo et al. 2019)
Ground corn & wheat extracts	AFB_1_ FB_1_	Solid state	SWCNT	0.46 pg/mL 0.34 pg/mL	89% – 98%	(He et al. 2024)
Wheat grain	DON, ZEA	E-nose	MOS array	nr	85‒93	(Borowik et al. 2024)

*nr* not reported

## Conclusion and future perspectives

Mycotoxin contamination in food and feed remains a significant global challenge, impacting public health, food security, and economic stability, particularly in developing regions. Conventional analytical methods such as LC-MS/MS, GC–MS, HPLC, and TLC offer high sensitivity, selectivity, and reliable quantification of mycotoxins. However, their reliance on complex sample preparation, expensive instrumentation, and skilled personnel limits their routine use for rapid, field-based monitoring. In response, nanomaterial-based sensor technologies have emerged as promising alternatives, offering rapid, sensitive, and portable detection across diverse food and feed matrices. Electrochemical, optical, piezoelectric, electronic-nose, and solid-state gas sensors each present distinct advantages depending on the application. VOC-based sensing allows for early, non-invasive detection of fungal contamination and serves as an effective screening method. However, these technologies are not yet replacements for established analytical methods; they are better positioned as complementary tools for preliminary detection.

Despite significant advancements, a major gap remains between laboratory-scale sensor development and real-world deployment. Key challenges include the lack of reproducibility in nanomaterial synthesis, where variations in particle size, morphology, and surface properties can affect sensor performance. Additionally, the cost of fabrication and functionalization, especially when using high-purity nanomaterials and biological recognition elements, limits large-scale production. Long-term stability and shelf life also remain concerns, especially under variable environmental conditions. Another critical barrier is the need for robust, user-friendly sensor formats suitable for field use. Many current systems rely on laboratory-based configurations or require controlled conditions, thus restricting their practical application. There is a demand for simple, reliable, and low-cost formats, such as disposable dipstick-type devices or portable integrated systems, that can operate effectively without specialized training. Furthermore, issues such as matrix interference, sensor drift, and a lack of standardized validation protocols continue to limit regulatory acceptance.

Future research should focus on improving the scalability and standardization of nanomaterial synthesis, developing cost-effective fabrication strategies, and enhancing sensor stability under real operating conditions. Integration with microfluidic platforms, wireless systems, and data-driven tools such as artificial intelligence can further improve sensitivity, automation, and real-time monitoring capabilities. Additionally, hybrid approaches that combine sensor technologies with confirmatory analytical methods will be essential to ensure reliability and support regulatory compliance.

## Data Availability

No datasets were generated or analysed during the current study.
